# SARS-CoV-2 variants: a new challenge to convalescent serum and mRNA vaccine neutralization efficiency

**DOI:** 10.1038/s41392-021-00592-6

**Published:** 2021-04-10

**Authors:** Maochen Li, Fuxing Lou, Huahao Fan

**Affiliations:** Beijing Advanced Innovation Center for Soft Matter Science and Engineering, College of Life Science and Technology, Beijing University of Chemical Technology, Beijing, China

**Keywords:** Vaccines, Infectious diseases

**Fig. 1:**
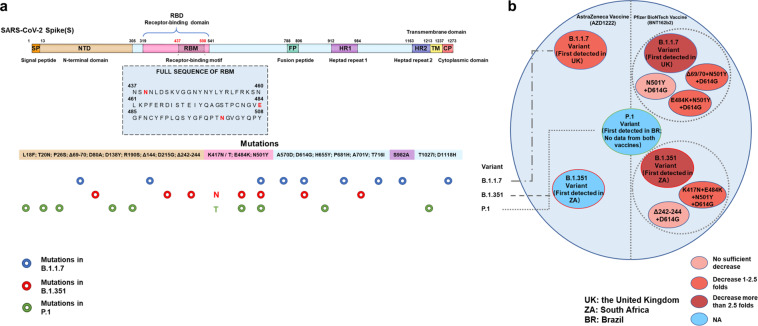
A schematic illustration of SARS-CoV-2 S protein sequence, prevalent mutations in current circulating SARS-CoV-2 variants, and the neutralization efficiency change of two mRNA vaccines—vaccine AZD1222 and vaccine BNT162b2. **a** RBM sequence (amino acid 437–508) containing N439K, E484K, and N501Y is a highly variable region, and SARS-CoV-2 spike protein mutations of three major circulating variants B.1.1.7, B.1.351, and P.1 are displayed, which can be tracked online (https://covariants.org/, https://cov-lineages.org/). **b** The neutralization efficiency of two major vaccines—vaccine AZD1222 and vaccine BNT162b2 to major circulating variants and potentially important variants of SARS-CoV-2 are exhibited. The neutralizing activity of the vaccine sera against different variants are shown in different colors from light red (no sufficient decrease), lycopene (decrease between 1.5-fold and 2.5-fold), to wine red (decrease more than 2.5-fold)

The spike protein of SARS-CoV-2 is the target of antibodies in convalescent and vaccine sera, and 23 mutations in spike protein were reported in the variants B.1.1.7, B.1.351, and P.1 (Fig. 1a). Recently, several groups evaluated the effects of convalescent and mRNA vaccine sera on two major circulating SARS-CoV-2 variants B.1.1.7 and B.1.351 (Fig. 1b),^1–5^ which leads to concerns about the immune escape of these variants from the human acquired immunity stimulated by previous infections and mRNA vaccines.

By 27 March 2021, COVID-19 has caused more than 126.1 million infections and 2,768,409 deaths (https://coronavirus.jhu.edu/), and some countries have already developed vaccines against SARS-CoV-2. Different from the inactivated vaccines approved in China, the western countries are prone to develop mRNA vaccines or viral vector vaccines (e.g., Oxford-AstraZeneca vaccine ChAdOx1 nCoV-19 (AZD1222) and Pfizer-BioNTech vaccine BNT162b2), targeting the spike protein, whose mutations deserve consistent monitoring. Because of the instability of SARS-CoV-2 RNA and error-prone replication, viral mutations appear frequently.^1,2^ Until now, 114, 67, and 36 countries have reported the discovery of variants B.1.1.7, B.1.351, and P.1, respectively (cov-lineages.org/global_report.html). Given these mutations may cause higher viral load and longer infection duration in the infected persons,^3^ the effects of B.1.1.7 and B.1.351 variants on virus infection and vaccine efficiency were studied by several groups, respectively (Fig. 1b).^1,2,4,5^

Supasa, P. et al. analyzed 180,000 sequences from the COG-UK database (https://www.cogconsortium.uk) and found that B.1.1.7 strain with amino acid 69 and 70 of spike protein deletion (Δ69/70) occupied the dominant position among the three subgroups (the other subgroups lack this deletion), which indicated selective advantages existed in the variation process of SARS-CoV-2.^1^ And N501Y mutation existing in all B.1.1.7, B.1.351, and P.1 variants were found to enhance the affinity between receptor-binding domain (RBD) and angiotensin-converting enzyme 2 (ACE2) by about 7-fold compared with wild type (WT). According to the data released by Public Health England, the infectivity of B.1.1.7 variant is 30–50% higher than that of the wild type (WT), which may result from the increased affinity between RBD and ACE2.^3^ Then the neutralization activities of 20 potent monoclonal antibodies for WT were detected by focus reduction neutralization tests, and a remarkable neutralization activity reduction was found in IGHV3-53 mAbs (e.g., mAb269 almost completely lost neutralization activity and mAb278 only retained 78% activity at most). In addition, the authors tested the efficacy of neutralizing antibodies in vaccine sera. The vaccine AZD1222 sera at 14 and 28 days following the second dose, and the vaccine BNT162b2 sera at 7–17 days following the second dose were tested, and the neutralizing activity of vaccine AZD1222 and BNT162b2 against the B.1.1.7 variant decreased by 2.5-fold and 3.3-fold, respectively. However, the cocktail therapy is still effective for neutralizing B.1.1.7 variant. Moreover, the convalescent sera from 13 B.1.1.7 patients can neutralize both B.1.1.7 variant and Victoria variant, indicating the potential of B.1.1.7 as the seed strain for future inactivated vaccines. Given the above information, the author hold the opinion that the current vaccines being arranged to massive scales against COVID-19 is still robust and no obvious evidence is found for immune escape.^1^

Almost at the same time, Peiyong Shi and colleagues estimated the neutralizing efficiency of BNT162b2 vaccine sera on N501Y mutants and B.1.351 variants.^4,5^ Based on the SARS-CoV-2 reverse genetic system developed previously, they obtained the variants using the SARS-CoV-2 USA-WA1/2020 strain, a isolate identified in January 2020: (1) N501Y; (2) Δ69/70 + N501Y + D614G; (3) E484K + N501Y + D614G; (4)Δ242-244 + D614G; (5) K417N + E484K + N501Y + D614G; (6) B.1.351-spike.^4,5^ The ratios of the neutralization geometric mean titers (GMTs) of the sera against above mutants to their GMTs against the USA-WA1/2020 virus were 1.46, 1.41, 0.81, 0.97, 0.66, and 0.37, respectively (Fig. 1b), which again implied that E484 and K417 are key sites for virus immune escape.^4,5^ Due to the continuous mutations of SARS-CoV-2 spike protein, mRNA vaccines might fail to have the potent efficiency as previously expected, suggesting viable methods (for instance, cocktail mRNA vaccines) are needed to be proposed to develop the effective mRNA vaccine.

Thomson et al. found that receptor-binding motif (RBM) sequence is a highly variable region, which contained N501Y mutation in both variants B.1.1.7 and B.1.351 mentioned above.^2^ And another mutation N439K, the second most prevalent mutation of spike protein also located in the RBM sequence (Fig. 1a), has been predicted to cause about 2–15 million infections worldwide according to a statistical model.^2^ N439K has been deemed to increase the viral load about 1.54-fold in 1918 Scottish patients, owing to the higher affinity between RBD and hACE2.^2^ Then, the recognition of N439K RBD by immune serum from 442 recovered individuals (6 patients were infected by N439K mutants) as well as 140 monoclonal antibodies from COVID-19 patients (including REGN-CoV, which obtained the EUA of FDA) were evaluated, 6.8% of the serum samples and 16.7% of mAbs had more than 2-fold reduction in binding activity, respectively. Interestingly, a camelid nanobody VHH-72 showed an increased neutralization on N439K variant, suggesting the mutation may be a double-edged sword for maintaining fitness of the N439K variants.^2^

In summary, these recent studies evaluated the major circulating SARS-CoV-2 variants B.1.1.7 and B.1.351 by convalescent and vaccine sera, and highlighted the importance of continuous SARS-CoV-2 molecular surveillance. Meantime, these researches are significant for the guidance of COVID-19 therapy, vaccine redesigning, and epidemic prevention: (1) For the COVID-19 patients, a personalized COVID-19 antibody therapy or cocktail therapy will be beneficial from the local circulating variants screening; (2) Desirable vaccine candidates, multivalent vaccines or cocktail vaccines should be designed to neutralize all circulating variants; (3) Inactivated vaccine seed strain of circulating SARS-CoV-2 variants is worthy of development for future epidemic prevention.
